# Socio-Epidemiological Factors with Negative Impact on Infant Morbidity, Mortality Rates, and the Occurrence of Birth Defects

**DOI:** 10.3390/healthcare9040384

**Published:** 2021-04-01

**Authors:** Elena Ţarcă, Solange Tamara Roșu, Elena Cojocaru, Laura Trandafir, Alina Costina Luca, Daniela Rusu, Viorel Ţarcă

**Affiliations:** 1 Department of Surgery II-Pediatric Surgery, “Grigore T. Popa” University of Medicine and Pharmacy, 700115 Iaşi, Romania; 2 Department of Nursing, “Grigore T. Popa” University of Medicine and Pharmacy, 700115 Iaşi, Romania; solange.rosu@umfiasi.ro; 3 Department of Morphofunctional Sciences I—Pathology, “Grigore T. Popa” University of Medicine and Pharmacy, 700115 Iaşi, Romania; 4 Department of Mother and Child Medicine–Pediatrics, “Grigore T. Popa” University of Medicine and Pharmacy, 700115 Iaşi, Romania; laura.trandafir@umfiasi.ro (L.T.); alina.luca@umfiasi.ro (A.C.L.); 5 Department of Surgery, “Grigore T. Popa” University of Medicine and Pharmacy, 700115 Iaşi, Romania; carmen-daniela.rusu@umfiasi.ro; 6 County Statistics Department, 700115 Iaşi, Romania; viorel.tarca@iasi.insse.ro

**Keywords:** chromosomal and genetic conditions, infant, morbidity, mortality, socioeconomic status

## Abstract

In the last 30–40 years, developed countries in particular, but also developing ones, have seen an increase in life expectancy and a decrease in infant mortality and morbidity rates. These factors are due to an increase in living standards, a decrease in differences between social classes, the increased accessibility of education to women, and the implementation of some public health measures. When certain basic social and medical measures are implemented on a large scale, their benefits are first reflected in lower infant mortality rates, and only in the second stage are such benefits reflected in decreasing neonatal mortality rates and a smaller number of stillbirths. In this study, we review the literature on these factors. We extrapolate and compare this literature with data recorded in our country in the hopes of finding the reasons why Romania ranks first in the European Union in terms of infant mortality rates. We found that lowering the infant morbidity, mortality, and congenital malformation rates is an absolute priority in Romania, which requires the involvement of decision makers in taking effective measures regarding food supplementation or enhancement using folic acid, adequate counselling of couples, monitoring of all pregnancies, setting antenatal diagnosis, implementing optimal delivery management and therapeutic approaches to problematic pregnancies in other hospitals and by involving the population in health education, avoiding occupational or in-home exposure to toxic factors, avoiding drug use, and implementing disease and infection prevention measures for pregnant women.

## 1. Introduction

In recent years, in both developed and developing countries, we have witnessed an increase in life expectancy and a decrease in infant mortality and morbidity rates (in the neonatal period) due to improved standards of living, fewer differences between social classes, better access to education for women, and the implementation of public health measures [1]. Some studies have shown a direct correlation between a mother’s education level and infant mortality [2]. When certain basic social and medical measures are implemented on a large scale, their benefits are first reflected in lower infant mortality rates, and only in the second stage do such benefits lead to decreasing neonatal mortality rates and a smaller number of stillbirths [3]. At the end of the 20th century, infant mortality amounted to about 4 million deaths each year, with about 99% of these deaths occurring in developing countries and almost half occurring at home [4]. After many alarms were sounded by various international organizations, and due to the implementation of mandatory minimal healthcare at birth, these figures have dropped by about two-thirds in only 15–20 years. However, infant mortality remains a major problem for healthcare systems in both developed and developing countries. About 1 million of the 2.7 million neonatal deaths that occurred worldwide in 2013 happened on the first day of life, and it was estimated that about two-thirds of these deaths could have been prevented by quality perinatal care [5]. In 2013, infant mortality amounted to 44% of the global mortality rate for children under 5 years of age, and in 85% of these cases, death occurred due to hypoxia during birth, perinatal infections, and prematurity or low birth weight [6]. It is estimated that approximately 50 million births (40% of all births worldwide) occur outside of hospitals under risky conditions for both the mother and the newborn [7]. The presence of birth defects is another important risk factor in increasing infant morbidity and mortality rates, with the prevalence of birth defects ranging from 1 to 4% worldwide [8].

## 2. Sources of Information and Aim of the Study

Using the Medical Subject Headings MeSH terms of chromosomal and genetic conditions, infant, morbidity, mortality, socio-economic status, and Romania, we performed a PubMed literature search for randomized controlled trials (RCTs), systematic reviews, observational studies, and case series from the earliest possible date to November 2020. We included articles published in English and also searched the reference lists for other relevant articles. In the present study, we review the literature on these factors and extrapolate and compare those previous studies with data recorded in our country in the hopes of finding the reasons why Romania ranks first in the European Union in terms of infant mortality rates.

## 3. Birth Defects and Causal Factors

The exact causes of embryonic development deficiencies and, consequently, birth defects are not exactly known since one defect may have several causes, and a single cause can produce multiple different malformations. Generally, 10% of these causes are genetic, another 10% are external (environmental factors), 20–30% are multifactor causes, and in about 50% of the cases, the cause is unknown. According to Laisk et al., about 15% of confirmed pregnancies end in miscarriage, with the causes of these miscarriages being largely due to chromosomal abnormalities inherited in the egg or spermatozoid that occur during egg development or spermatogenesis; other causes may include the action of some toxic factors (smoking, alcohol) after the egg has been fertilized or some maternal diseases [9]. Some studies show that 40–60% of all fertilized human eggs are lost within the first three weeks post-conception, and 50–60% of miscarried fetuses have chromosomal abnormalities [10]. 

For a defect to occur, the causal factors must intervene in the critical period of the relevant organ that is not concomitant with the critical period of other organs. If the same causal factor impacts the same organ in a period other than the critical one, that factor is no longer capable of impeding the organ’s normal development. Thus, each organ has its own critical period, and each teratogenic agent has a predilection for a particular organ. The critical period corresponds to embryonic growth and differentiation, which are thought to occur between the 15th and 60th days after fertilization [9]. If the teratogenic agent acts within the first two weeks, it may directly cause the death of the blastocyst or the embryonic disc, and if it acts after the first 14 days, that agent may cause serious malformations, chromosomal abnormalities, or miscarriage [10]. In the absence of proper family planning, in which the pregnancy is scheduled and the future parents lead a balanced life and resort to the periconceptional supplementation of folic acid and other vitamins, pregnancy may occur, and the embryo may begin to develop without the mother’s awareness [11]. The mother lack of awareness regarding the pregnancy may subject the fetus to the action of some toxic factors during its most vulnerable period, namely, during the embryogenesis period, with the appearance of chromosomal abnormalities, malformative syndromes, or miscarriage. Therefore, social education and family planning are extremely important factors in determining stillbirth, as well as neonatal and infant mortality rates, and should be implemented and promoted in the family—first and foremost, among the young population, starting from primary education, when children learn what a healthy and balanced life means.

We have recently witnessed, both worldwide and in our country, an increase in the incidence of chromosomal abnormalities and birth defects, as well as an increase in the rate of prematurity and low birth weights, all of which have many causes [12]. This higher incidence of birth defects may be accounted for by changes in demographic factors such as the young ages of parents, especially mothers; inbreeding; extensive smoking and cocaine use; the use of other vasoconstrictive pharmacokinetic agents during the gestational period; and radiation or other environmental factors [13]. A relatively recent study showed that despite a two-year increase in the average age of mothers, in parallel with the decrease in smoking rates from 34% to 22% in the same population, an increase has been observed in the incidence of abdominal wall malformations in the fetus, likely under the influence of factors other than those analyzed [14].

A European study conducted over a 10-year period analyzed the ages of mothers of children with omphalocele and detected a higher risk for this condition among young mothers under 20 years of age compared to mothers aged 25–29, with this risk being higher for isolated cases of omphalocele than for omphalocele associated with other defects [15]. An older maternal age also seems to be a risk factor for omphalocele, which is accounted for by the increase in the incidence of chromosomal abnormalities when the mother is over 35 years old, with 30% of chromosomal abnormalities associated with omphalocele [16]. For omphalocele, miscarriage is closely related to chromosomal abnormalities, abortion affecting 10–24% of all such pregnancies. The occurrence of the miscarriage of fetuses with laparoschisis, another malformation of the anterior abdominal wall, is also not negligible, affecting up to 23% of pregnancies, especially during the last trimesters of the pregnancy [17]. The causes of these intrauterine deaths remain incompletely elucidated, but the onset of intestinal complications such as sudden dilation or perforation of the loops may explain some miscarriages. Therefore, some authors recommend the careful monitoring of problematic pregnancies via home monitoring and frequent ultrasound scans in the last trimester of pregnancy to promptly detect possible fetal suffering and perform an emergency caesarean section if necessary [18]. Other studies have demonstrated that oxidative stress is present in pregnancy complications and that low plasma antioxidant status is associated with the subsequent development of pregnancy complications [19].

Another example is bladder exstrophy, for which no single teratological agent or environmental factor could be identified. Nevertheless, according to a study by Reutter et al. on a large cohort of patients and their families, smoking and medical radiation in the first trimester of pregnancy could cause a more severe expression of the defect [20]. The old age of the parents, multiparity, and in vitro fertilization are considered causative factors [21]. Isolated reports highlight alcohol consumption and some drugs such as phenobarbital, valproic acid, diazepam, heparin, and misoprostol as teratogenic factors that play a role in bladder extrusion, cranio-facial deformities, and cardiac or neural tube defects [22]. A certain number of teratogenic factors are recognized as being responsible for cranio-facial malformations. The most important teratogenic factor that causes cranio-facial malformations—and probably the best studied—is alcohol. Drugs, such as hydantoin and accutane, may also cause cranio-facial abnormalities in humans, just like toluene, tobacco, ionizing radiation, and hyperthermia [23]. It has also been found that children from diabetic mothers or those working in the leather industry (with organic solvents) have an increased risk for such cranio-facial abnormalities. Holoprosencephaly may also occur within chromosomal abnormalities such as trisomy 13, 18, and 21. Thus far, the exact mechanisms by which drugs, chemicals, and other environmental factors influence the development of the embryo and induce abnormalities remain unknown. Even the mechanism of action for thalidomide (one of the most well-studied and well-known teratogenic factors) on the embryo remains a mystery, with over 20 hypotheses proposed to date on how thalidomide impedes embryo development [24].

Many other drugs, such as antibiotics, anti-emetics, benzodiazepines, sulphonamides, oral contraceptives, anti-inflammatory drugs, and antipyretics, as well as periconceptional exposure to certain toxic solvents or dyes, herbicides, and X-rays, have been studied for their connection with the occurrence of anterior abdominal wall defects in newborns. Although the results of these studies are sometimes contradictory, most of them demonstrate a directly proportional connection [25,26,27]. Chemical compounds used in agriculture and found in drinking water may also have teratogenic effects on pregnancy. The presence of atrazine, arsenic, or nitrates may lead to neural tube defects, abdominal wall defects, limb abnormalities, or other birth defects.

## 4. Parents’ Socio-Occupational Status and Lifestyle

The parents’ origins may also play an important role in the occurrence of certain birth defects due to the action of toxic environmental factors on pregnancy during embryogenesis. Pollution specific to residential areas near landfills or in large heavily industrialized urban agglomerations has led to higher birth defect incidence rates in some cities in Finland, USA, UK, and Poland [28,29]. Low socio-occupational status is also frequently associated with exposure to toxic environmental factors such as pollution in heavily industrialized areas or near major highways; in this context, the more frequent occurrence of cardio-vascular and pulmonary malformations in association with high levels of carbon monoxide in atmospheric air has been noted [30]. 

A rural environment is usually associated with a low socio-economic and cultural level and to some old customs that are not safe from a healthcare perspective. Commonly in rural areas, and increasingly often in urban areas, births take place at home, under dangerous conditions from the perspectives of the people who attended the birth, hygiene, thermal comfort, and mother–newborn safety. Observing several basic neonatal care principles, such as delivery under hygienic conditions, cutting and dabbing the umbilical cord with antiseptic solutions, ensuring that the baby’s upper airways are clear, ensuring the thermal comfort of the newborn, and encouraging breast feeding are simple but essential requirements for newborn survival that should be accessible in any environment where a delivery will take place [31,32]. A simple evaluation of newborns and the provision of a minimum level of healthcare could reduce the perinatal mortality rate due to so-called ‘asphyxiation at birth’ by at least 10% [33]. All newborns require a minimum level of assistance at birth to begin breathing and adapting to the transition to extrauterine life, thus decreasing neonatal morbidity and mortality rates via simple maneuvers that can be performed by any skilled individual under minimum hygiene conditions and with no specialized equipment; 5–10% of newborns require skin drying, heating, and stimulation (via back rubbing with a clean towel or by flexing the lower limbs), and only 1–3% require resuscitation in order to breathe, e.g., through mask ventilation, chest compression, orotracheal intubation, or specific medication. A previous studied showed a 60% reduction in neonatal mortality in a rural community in India by simply educating women in the village on minimal healthcare during home delivery [34].

Studies provide conflicting data regarding the influence of recreational drugs (e.g., crack, ecstasy, amphetamines, marijuana, and LSD) on the occurrence of abdominal wall defects in newborns [35,36]. The connection between the mother’s use of cocaine and the occurrence of laparoschisis in the fetus is of particular interest because cocaine is a vasoconstrictor, and this action mechanism is often observed in the etiopathogenesis of laparoschisis. This theory is also supported by some studies that found an increased rate of laparoschisis among children born to mothers who used vasoactive medication, such as salicylates, pseudoephedrines, and phenylpropanolamines, during pregnancy [25,37]. 

## 5. Recommended Nutrition during Pregnancy

A balanced diet and the periconceptional use of vitamins, especially folic acid and vitamin B12, by both the mother and father may reduce the risk of neural tube defects, abdominal wall malformations, and heart malformations, according to several studies [38,39,40]. The efficacy of folic acid in preventing the occurrence of omphalocele or neural tube defects has been demonstrated, but the preventive effect of this treatment is increased if folic acid is taken three months before conception and continued throughout the first pregnancy trimester [41]. Since this is not always feasible at the population level, especially in low-income countries, where more than half of the total number of pregnancies are unplanned, another method that has already begun to be implemented is industrial food enhancement using folic acid [42]. Food enhancement using folic acid may prevent about 46% of neural tube defects and reduce the neonatal mortality rate due to visible birth defects by 13% [42]. Good health and nutrition before conception are central to a mother’s ability to meet the nutrient demands of pregnancy and breastfeeding and are vital to the healthy development of the mother’s embryo, fetus, infant, and child. Many women and adolescent girls are malnourished because of the inadequacy or imbalance of their diets, leading to malnutrition and micronutrient deficiencies or, conversely, overweight and obesity [43]. Particular attention should be paid to the intake and status of some micronutrients in women of reproductive age, especially folate, but dietary supplementation with iron, zinc, vitamin D, vitamin B_12_, iodine, and others may also be recommended for women at risk of the poor supply and insufficiency of these micronutrients. The importance of vitamin D for fetal skeletal development is well-known, and maternal deficiency results in a low birth weight, increased risk of neonatal hypocalcemia, cardiac failure, osteopenia in the newborn, and childhood rickets [44]. Pregnant women should consume a balanced diet and should not increase their dietary energy intake during late pregnancy by more than about 10% above the recommended energy intake for non-pregnant women to avoid obesity [43]. Healthy pregnancy outcomes are more likely if the woman who enters pregnancy is physically active, has a healthy diet, does not smoke, avoids alcohol, and has a normal body mass index.

## 6. Antenatal Diagnosis and Therapeutic Abortion

An increasing number of newborns with birth defects benefit from antenatal diagnosis. For this reason, whenever an obstetrician finds a fetal anomaly during ultrasound scanning, a multidisciplinary antenatal consultation should be offered to the parents [45]. The purpose of this antenatal consultation when congenital abnormalities or malformations are detected is to provide information on the progress of the pregnancy and the fetus’ quality of life, possible ante- or postnatal procedures, appropriate management and type of delivery, and long-term prognosis. If other related abnormalities are detected or if the malformation falls within a certain genetic syndrome, the fetus’ prognosis may be poor, and the parents may consider elective termination of the pregnancy [11]. This decision varies greatly depending on the antenatal diagnosis team, the attitudes of the parents, the state of the fetus, and the therapeutic abortion legislation in force at the time. The parents’ religious beliefs or socio-economic and educational status may also have an important influence on therapeutic abortion decision-making. In such cases, parents should be given the opportunity to discuss the fetus’ prognosis with the neonatal team members and a specialized psychologist to fully assess the situation and free themselves of fear or guilt before making the final decision. Due to all these factors, the therapeutic abortion criteria are difficult to define and harmonize worldwide, which accounts for the significant differences in related figures found in the literature. Some studies show that 60% of birth defects are preventable via relatively simple measures and that about 80% of all severe abnormalities can be detected before birth via routine fetal examinations, which may give the couple the possibility to benefit from antenatal counselling and decide whether to resort to therapeutic abortion [11,46]. These measures would obviously lower the rate of congenital malformations present at birth, as well as the infant mortality rate, as over 25% of neonatal deaths are due to major congenital abnormalities in both developed and developing countries [47].

## 7. Preventive Measures to Reduce Perinatal Mortality and Long-Term Morbidity

Pregnancy and childbirth represent a critical window of opportunity for providing effective interventions to prevent preterm births and other adverse health outcomes associated with an early birth. These interventions include the provision of needed social and financial support to disadvantaged mothers, as well as workplace, professional, and other supportive policies promoting safe motherhood and women’s universal access to care before, during, and after pregnancy [48]. The administration of antenatal corticosteroids to pregnant women at high risk of preterm births as early as 23 weeks can significantly reduce a premature infant’s risk of death, respiratory distress, and developmental problems [49]. Antibiotic treatment for preterm premature ruptures of the membranes has been shown to delay the onset of labor for up to 48 h and reduce neonatal infections, as well as abnormal cerebral ultrasound scans prior to hospital discharge [50]. Behavioral and community-based interventions, which can lead to reductions in smoking and violence against women, need to be implemented in conjunction with antenatal care models that promote women’s empowerment as a strategy for reducing preterm delivery [48]. There are other simple services that can also be performed during the pregnancy period with a high impact on reducing preterm birth rates and mortality rates, such as identifying women at high risk of preterm birth; the screening for and treatment of sexually transmitted diseases, including HIV and other infections (tuberculosis, malaria, bacterial vaginosis, and bacteriuria); the correction of malnutrition; micro-nutrient supplementation; and the cessation of smoking, alcohol, and other drugs [48]. Immunization in pregnancy is another promising strategy to reduce infectious disease-related morbidity and mortality in pregnant women and their infants. Vaccines against tetanus, pertussis, and seasonal influenza have been recommended for the routine immunization of pregnant women in high-income countries and in some low and middle-income countries (LMIC) for many years and have been determined to be both safe and effective at preventing infections. Despite a related recommendation by the WHO, influenza immunization for pregnant women has not been incorporated into immunization programs in many LMIC [51]. Unfortunately, 1.8 million children die within the first month of life, with many deaths due to infections that have the potential to be prevented through existing vaccines or vaccines under development for delivery to pregnant women [52]. New vaccines for administration to pregnant women are currently under development, such as respiratory syncytial virus, group B streptococcus, cytomegalovirus, and monovalent pertussis vaccines [51].

The world has recently witnessed an increase in the incidence of chromosomal and genetic abnormalities, conditions that also increase the incidence of global developmental delays and intellectual disabilities, which are two main clinical subtypes of neurodevelopmental disabilities (NDDs). Although concrete NDD etiologies could not be identified in 48.4% of patients, genetic diseases (comprising a proportion of 35.8% of total cases), including inborn errors of metabolism and congenital dysmorphic diseases, constituted the most common etiology category for NDDs [53]. Prematurity is another cause of NDD. In this regard, the administration of magnesium sulphate to women at risk of preterm birth helps to protect the infant’s brain, reduces rates of cerebral palsy, and improve long-term neonatal health outcomes [48]. Over the last 50 years, almost all European countries have introduced neonatal screening for metabolic diseases as an important public health feature. There are large variations in the panel of screened conditions, ranging from 0 to more than 30 conditions in different states. There is a need to further improve access to developing countries in the implementation of fundamental screening programs for disorders such as PKU and congenital hypothyroidism. All screening in public health is performed to achieve health gains, but in Europe, the results are such that in some—mostly smaller—countries, considerable changes have been implemented, mainly concerning the number of ms/ms (tandem mass spectrometry)-detectable conditions. In contrast, in other—mainly larger—countries, very little has changed, if at all [54]. In Table 1, we summarize the main factors responsible for neonatal morbidity and mortality, as well as possible preventive health measures.

## 8. Neonatal Morbidity and Mortality Rates in Romania

**Figure 1:**
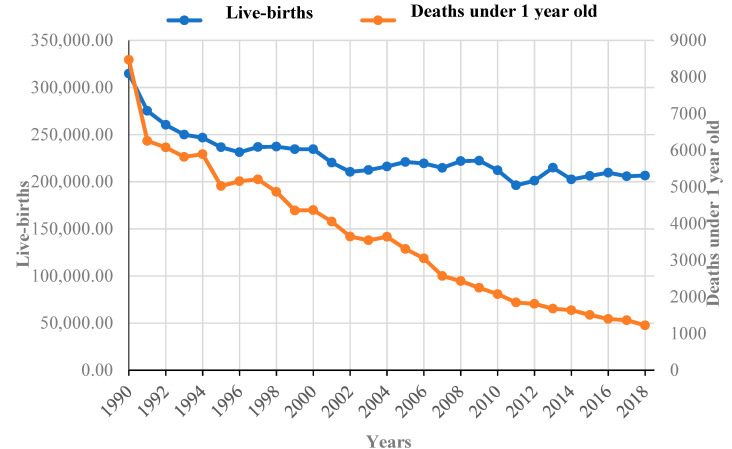
Evolution of the number of live births and of the number of deceased persons under the age of 1 in Romania between 1990 and 2018 [56].

Unfortunately, in Romania, there are significant differences between rural and urban areas, as well as between different social categories regarding their access to healthcare services, public education, and both infant and mother mortality and morbidity rates. For example, in 2012, the mortality rate among children under one year of age was 11.8‰ in rural areas, compared to 6.6‰ in urban areas; rural areas were also unfavorably associated with higher home death rates and not going to the general practitioner (GP) in the case of infant respiratory diseases [55]. The status of deaths among children under one year of age due to various causes reveals that birth defects, respiratory diseases, and perinatal disorders are the main causes of mortality. Thorough monitoring and disease prevention measures may have considerable effects in lowering mortality rates. The birth rate has dramatically decreased in our country in recent decades, by 1.5 times between 1990 and 2018 (the primary data were taken from the official website of the National Institute of Statistics and processed by the authors in Figure 1 [56]). However, an encouraging fact is the even more marked decrease (almost 7 times) in the infant mortality rate over the same period. This indicates that, especially since 2002, the drastic decrease in the number of deceased persons under the age of 1 alongside the virtually unchanged number of live births is due to breakthroughs in the Romanian medical scientific world, as well as the high degree of professionalism of staff directly involved in providing good quality services to the population in our country.

Between 20,000 and 24,000 premature children are born in Romania each year; prematurity and low birth weight are important risk factors in infant mortality, with the mortality rate reaching 48.6‰ among premature infants weighing less than 2500 g compared to 3.6‰ among those weighing more than 2500 g [55]. 

Until 1989, Romania was a communist country, and its healthcare system was characterized by centralized planning and severe underfunding with low-performance and low-quality healthcare. Since then, some political changes have yielded improvements in life quality and the healthcare system, especially after Romania joined the European Union in 2007 [57]. From a child healthcare point of view, there have been consultations for neonate and infant care at home, periodical medical examinations up to the age of 18 years, counseling and administration of vaccines according to the National Immunization Program alongside optional vaccines for children, health education, and the identification of risk factors, which are priority activities for pediatric family physicians. The public health system has 11 pediatric hospitals, one institute for mothers and childcare in Bucharest, and three rehabilitation hospitals for children. In most of the counties and cities of Romania, single-specialty or private hospitals have a pediatric department [57]. Since 2002, the public medical system for neonates was reorganized to improve the quality of health care. According to the National Institute for Statistics, in 2016, there were 7666 pediatric beds, 4630 beds for neonates and premature babies, and 535 neonatal intensive care unit (NICU) beds in maternity units in Romania [56]. The SMURD system (Mobile Emergency Service for Resuscitation and Extrication, i.e., Serviciul Mobil de Urgenta, Reanimare si Descarcerare in România) is a complementary rescue system that is integrated with the national emergency system and can be accessed using an emergency call number. This system addresses the most difficult cases and also includes a helicopter emergency medical system [57]. 

The efforts made by the government and healthcare professionals over the last two decades to raise the economic level of the country and ensure the survival of premature newborns reflect normal attitudes in a responsible and civilized human society and feature directly visible effects—namely, a decrease in the infant mortality rate from 24 deaths in one thousand births in 1994 to 9‰ in 2012 and 6.4‰ in 2018 [55,56,58,59]. Despite these positive developments, infant mortality remains a major problem in our country, as Romania ranks first in the European Union in this area, with an infant mortality rate of 6.7‰ compared to an average of about 3.6‰ in 2017 [56]. Nevertheless, according to the information provided by Eurostat, the statistical office of the European Union, over the course of ten years, Romania has made the most significant progress in reducing its infant mortality rate [56].

An important and encouraging fact is that most deaths are due to perinatal causes and respiratory diseases. These causes of mortality can be prevented by the adequate involvement of decision makers. The deaths caused by diseases of the respiratory system are below 2‰ in developed countries, whereas in our country, this index was 2.6‰ in 2012 (the second cause of death in the <1 year age group, i.e., 29% of all deaths) [55]. The rate of deaths due to congenital abnormalities, which was 2.1‰, may also be lowered by antenatal diagnosis setting and elective pregnancy termination in the case of fetuses with multiple congenital abnormalities or severe chromosomal abnormalities and, in the case of congenital malformations that can be corrected before or immediately after birth, by scheduling and assisting the delivery and immediately taking the newborn to a specialized pediatric surgery center. The mother’s age group is also a risk factor for infant mortality, with the most vulnerable age groups being under 20 and over 40 years old. The mother’s education level is also inversely proportional to the infant mortality rate: 12.8‰ deaths for mothers with primary education (completed or not) compared to 3.3‰ for mothers with higher education [55]. The lack of education and information among the population regarding the necessity of health insurance and the poor accessibility to health services cause some potential applicants to remain outside the public insurance system. A low education level also relates to a lack of insurance and high morbidity and mortality [59].

Studies on anterior abdominal wall malformations conducted on 219 cases in Romania over a 20-year time span showed a 42.1% prematurity rate and a 64.9% low birth rate for laparoschisis. As far as omphalocele is concerned, 28.5% of newborns were premature and 35.2% had a low birth weight. Unfortunately, 23% of laparoschisis pregnancies and 26% of omphalocele pregnancies were not monitored by a GP, and only 11.4% of laparoschisis cases and 13.3% of omphalocele cases were diagnosed before birth [12,60]. It should also be noted that 40% of the mothers of children with laparoschisis were unmarried compared to 22% for omphalocele, while the parents’ alcohol and tobacco consumption rates were 15% in the case of laparoschisis compared to 32% for omphalocele; 41.2% of the mothers of children with laparoschisis were under 20 years old, and 27.6% of the mothers of children with omphalocele were over 30 years old. In total, 68.5% and 62%, respectively, of the children with laparoschisis and omphalocele were born to mothers from rural areas. The antenatal diagnosis rate was doubled for newborns from urban areas compared to those from rural areas, which is statistically significant. All these socio-economic factors negatively influenced the addressability rates for mothers of children with anterior abdominal wall defects and the rates of antenatal diagnosis, which had a negative impact on the subsequent evolution of these cases. This low antenatal diagnosis rate led to delayed presentation of the newborn to specialized healthcare units, as well as delays in appropriate surgical procedures and subsequent monitoring in the neonatal intensive care unit, making the newborn more prone to infections. Even in the Romanian healthcare system, which is characterized by many shortcomings and material deficiencies, antenatal congenital malformation diagnosis is associated with positive results, including a 61.5% survival rate for laparoschisis patients diagnosed before birth versus 25.7% in the absence of antenatal diagnosis [12]. In Romania, surgically correctable congenital malformations are the main cause of infant morbidity and death in children under one year of age [55]. Unfortunately, socio-cultural, educational, demographic, and economic factors limit the accessibility and quality of pediatric surgery services in our country. Some studies show that the true human and financial costs of congenital abnormalities are largely underestimated. Pediatric surgery is a cost-effective solution with significant potential to prevent premature mortality and long-term disabilities [61]. 

Given the global efforts aimed at combating healthcare disparities, we also attempted to identify and combat the factors that lead to these differences in Romania. Better involvement of civil society, the education and healthcare systems, and the authorities in health education and general disease prevention measures, as well as the endowment of maternity hospitals and pediatric surgery units with proper equipment, sufficient budgets, and properly trained healthcare professionals, will undoubtedly lead to a significant reduction in the infant mortality rate.

## 9. Conclusions

Lowering the infant morbidity, mortality, and congenital malformation rates is an absolute priority in Romania and requires decision makers to take effective measures regarding food supplementation or enhancement using folic acid, adequate counselling of couples, monitoring of all pregnancies, setting antenatal diagnosis, and developing the best delivery management and optimal therapeutic approaches for problematic pregnancies in other hospitals, as well as involving the population in health education, avoiding occupational or in-home exposure to toxic factors and drug use, and deploying disease and infection prevention measures for pregnant women.

## Figures and Tables

**Table 1:** Key factors of neonatal morbidity and mortality and possible preventive health measures.

Key Factors of Neonatal Morbidity and Mortality	Lack of Sexual Education and Primary Care Education	Parents’ Socio-Occupational Status	Chromosomal Abnormalities, Birth Defects, and Metabolic Diseases	Miscarriage and Intrauterine Deaths	Perinatal Infections
**Possible preventive health measures**	- sexual education and family planning - primary education, where children learn what a healthy and balanced life means, with long-term consequences - promoting a healthy lifestyle, starting with the primary education of children and continuing with the involvement of the church and the media	- reducing the pollution specific to residential areas - implementation of simple but essential requirements for hygiene and newborn survival - educating women in rural areas on minimal healthcare for at-home deliveries - social and financial support for disadvantaged mothers	- proper family planning - avoiding the consumption of toxic foods, alcohol, tobacco, and drugs - periconceptional supplementation of folic acid, vitamin B12, and other vitamins - maintaining a normal body mass index - neonatal screening for metabolic diseases - improving access to medical services and improving the rate of the antenatal diagnosis of malformations	- careful monitoring of problematic pregnancies - home monitoring and frequent ultrasound scans - administration of antenatal corticosteroids to pregnant women at high risk of preterm birth - emergency caesarean section if necessary	- antibiotic treatment for preterm premature rupture of the membranes - screening for and treatment of sexually transmitted diseases including HIV and other infections - immunization during pregnancy
